# Influence of Pulsed Arc Parameters on the Tig Welding Process for the Stainless Steel Duplex UNS S31803

**DOI:** 10.3390/ma16216870

**Published:** 2023-10-26

**Authors:** Vinicius Marques Alves Generoso, Lucas Menezes de Souza, Elaine Cristina Pereira, Sergio N. Monteiro, Afonso R. G. de Azevedo

**Affiliations:** 1 UENF—State University of the Northern Rio de Janeiro, LAMAV—Advanced Materials Laboratory, Av. Alberto Lamego, 2000, Campos dos Goytacazes 28013-602, RJ, Brazil; viniciusmarques84@gmail.com (V.M.A.G.); 202011220016@pq.uenf.br (L.M.d.S.); elainecp@pq.uenf.br (E.C.P.); 2 IME—Military Institute of Engineering, Materials Science Program, Praça General Tibúrcio, 80, Urca, Rio de Janeiro 22290-270, RJ, Brazil; snevesmonteiro@gmail.com; 3 UENF—State University of the Northern Rio de Janeiro, LECIV—Civil Engineering Laboratory, Av. Alberto Lamego, 2000, Campos dos Goytacazes 28013-602, RJ, Brazil

**Keywords:** stainless duplex steel, microstructure, post weld heat treatment, cyclic ratio, pulsation frequency

## Abstract

The influence of parameters involved in the pulsed electric arc, used as an energy source in the tungsten inert gas (TIG)-mediated welding of Duplex UNS S31803 stainless steel, to attend the manufacture of flexible pipes for the extraction of oil and gas is presented. A fundamental part in the manufacturing process of flexible pipelines is the welding of these strips so that corresponding TIG welds will be subjected to the same process and work conditions. Therefore, it is necessary to maintain the same properties in the welded regions. Covering the effects of each parameter of the pulsed electric arc such as peak and base current as welds, cyclic ratio, and pulsation frequency is a desirable endeavor. The final objective is the mitigation of problems that have a great impact on production, such as weld breakage during the conformation of the strip in the process and test failures. With this, tensile, bending, and ferrite percentage tests were performed on 12 samples that qualified as satisfactory in the visual aspect. A minimum tensile strength of 734.57 MPa and a maximum of 775.77 MPa were obtained where all values found are above the tensile strength limit of the base material of 620 MPa. With the completion of the study, it is possible to understand not only the response of the process to each parameter but also the tendency when changing them. Moreover, it is possible to explore the possibility of guiding the changes to achieve results about the visual aspect and the mechanical properties of the welded material.

## 1. Introduction

Oil and gas extraction from deep ocean waters is demanding increasingly unique technologies and materials. As an important resource which has been widely used for the transport of fluids and instrumenting systems on offshore platforms, flexible pipelines are complex structures with multiple layers whose number and type depend on the specific design requirements [1,2].

These pipelines use helical reinforcement of steel wires and strips combined with concentric layers of polymers, textile tapes, and adhesive tapes, culminating in a structure capable of withstanding considerable structural loads as well as internal and external pressures. The target of the present study, the inner layer of the flexible pipeline, which has contact with the corrosive fluid composed of oil and saline water, must be made of corrosion-resistant steel. Therefore, in its manufacture, metallic strips of stainless steel of different classes are used including the UNS S31803 steel depending on the severity of the fluid to be transported. In the specific case of oil, it will depend on the type of productive well, particularly corrosive aspects of the oil withdrawn from this well [1,2].

The manufacturing processes for these pipelines are established in forming and welding sequences. Several layers make up the flexible pipeline structure. The initial structure, made up of duplex stainless steel, mostly flexible pipelines, is fitted with a pre-formed “tape” fitting system to allow this coupling and assembly as a “spiral”. For the “profiling” process to maintain its continuity, strips of the steel in question are welded together, maintaining a continuous system until the desired length of the pipeline is obtained. Depending on the severity of the forming process, the joints must have good mechanical characteristics. This conformation consists of the “S” shape of the duplex stainless steel “tapes”, for a sequential fit and “profiling”, thus achieving what is known as a flexible pipeline. Due to the complexity of the structures produced, some regions of the generated joint may be potential sites of structural problems. This becomes more serious the greater the complexity of the alloy to be welded [1,2,3].

In addition to all requests during the manufacturing process of these pipelines, in field conditions they are subject to several constraints, among which it is worth mentioning the tensile loading of the entire assembly. In addition, compression loads can be experienced during installation in deep water, namely a buffer effect due to high hydrostatic pressure, or, in extreme situations, such as when the floating unit operates in low top angle conditions and is subject to sudden movements [1,4]. This whole complex loading system leads to a greater concern about the quality of the materials and process in use. For each pipe manufactured, several welds are performed and such unions are normally carried out through the tungsten inert gas (TIG) welding process, which has the electric arc as an energy source. TIG might be configured to provide the input of heat needed to promote the fusion of the tape with different nuances [5,6,7,8,9].

UNS S31803 duplex stainless steel is an alloy based on the Fe–Cr–Ni system. Its chemical composition and thermomechanical processing give it a biphasic microstructure with approximately equal proportions of ferrite and austenite. It typically has relevant Cr and Ni values, with very low carbon contents (less than 0.03%) and with additions of nitrogen and Mo [9,10,11,12]. Table 1 shows the percentage by weight of the alloying elements of the reference material which are the same for the sample material.

UNS S31803 duplex stainless steel has numerous advantages over traditional austenitic and ferritic stainless steels. Its mechanical strength is approximately twice that of common austenitic stainless steels, combined with good toughness. It has high resistance to stress corrosion cracking and localized corrosion in environments containing chlorides [13]. Its weldability is superior to that of ferritic stainless steel. The hardness of the base material is dependent on the type and level of cold work [14,15,16,17].

Regarding the fraction of austenite and ferrite, it is verified, according to the current literature, that the adjustment of the ferrite/austenite balance during the manufacture of these steels is established at temperatures in the order of 1000 °C [18] and that such balance is situated at around 50%. The literature shows that the welding thermal cycle has a great influence on this balance, verifying that the greater the heat flux in the cooling process, the greater the ferrite content, thus causing a greater imbalance between the phases [6,10,12].

The application of duplex stainless steels in the petrochemical sector has led to the elaboration of quality criteria for this type of steel and for welded joints, both in terms of macro and microstructure, mechanical properties, and corrosion resistance, with special attention to the fraction of ferrite/austenite. The definition of these criteria requires proper control of the transformation processes involving such materials, especially welding processes, due to the heat cycles that such processes affect the metal during the execution of welds [5,19,20].

The TIG process uses an electric arc maintained between a non-consumable W electrode and the piece to be welded as a heat source. The welding region is protected by an inert gas flow. Welding can be performed with or without filler metal and can be manual or automatic. Currently, the TIG process is most used in the welding of Al, Mg, and Ti alloys and stainless steels. The solder produced is of very good quality [6,7].

**Figure 1:**
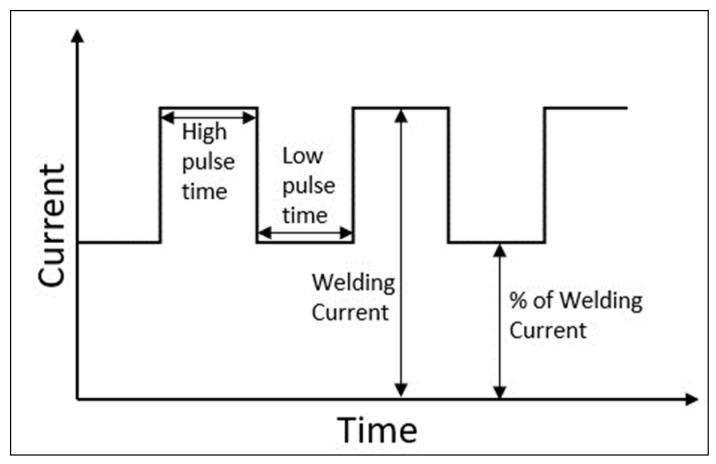
Current graph for the pulsed source.

The TIG welds are autogenous, without added material, and were performed through a semi-automatic process where the movement of the torch was automated, providing repeatability and standardization. A pulsed direct current was used, which is characterized by an intensity variation in a minimum value (base current) and a maximum value (peak current). The type of wave used is the square wave, as shown in Figure 1, where the values of intensity and the duration of peak and base currents and pulse frequency must be established [6,8,21].

In order to systematize the elaboration of the settings, it is necessary to characterize the influences of the settings relevant to the electric arc pulse produced by the welding machine generator. This is an objective that technically made the proposed research project feasible.

The main advantage of pulsed current is that it allows a combination of power, good penetration, and pulse fusion while keeping the weld area relatively cool. Thus, it is possible to obtain greater penetrations than in constant direct current and to work with materials that are more sensitive to heat input with minimization of distortions of the base metal (BM). Rectangular wave pulsed current is characterized by the periodic alternation between high and low levels of welding current at a given frequency. This wave configuration allows the arc energy to be used more efficiently, bringing benefits such as greater control over the dimensional characteristics of the weld bead, greater tolerance to variations in heat dissipation, less heat input, a reduction in residual voltages and distortions in the parts as welds, greater control over the weld pool, grain refinement in the fusion zone (FZ), and a reduction in the width of the thermally affected zone (HAZ) [22,23].

This article presents a study developed to evaluate the influence of the parameters of the pulsed electric arc used as an energy source in the TIG process applied to the welding of Duplex UNS S31803 stainless steel (DSS). In total, 27 different weld settings were proposed and the visual appearance and mechanical and metallurgical properties were evaluated.

## 2. Materials and Methods

**Figure 2:**
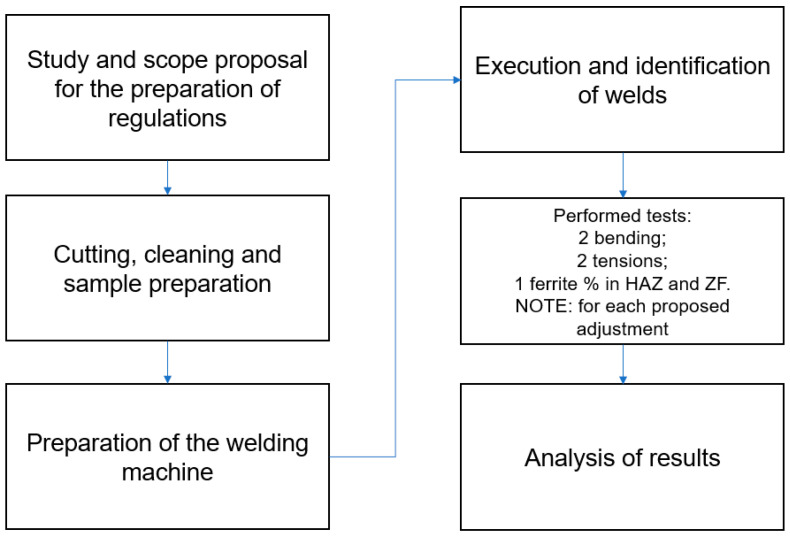
Experimental steps.

The DSS strip went through the hot lamination processes with subsequent treatment followed by air cooling, until reaching the final thickness of the plate. After cutting, plates have a width of 108 mm and a thickness of 2.7 mm. The chemical composition in percent by weight of the DSS strip UNS S31803 is presented in Table 1 and the mechanical properties in Table 2 [14,24]. Figure 2 shows a schematic of the experimental stages of this research.

For the execution of the welds, the semi-automatic TIG welding machine with a torch with automated displacement, Oerlikon CITOTIG II 300 DC, was used.

Therefore, 27 adjustments were proposed and elaborated through a combinatorial analysis where the alteration of each variable implied the maintenance of the others. According to Tables 3 and 5 welds were performed for each one of these adjustments.

**Figure 3:**
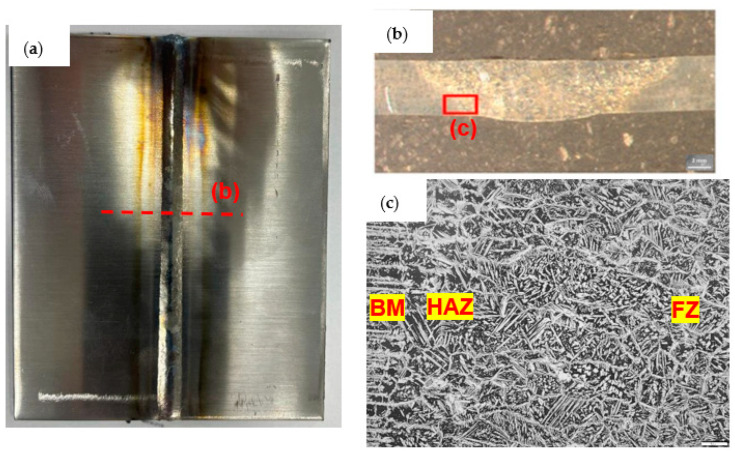
(**a**) Weld image at 10×; (**b**) Weld image at 50×; (**c**) 200× image with definition of the weld regions (region highlighted in (**b**)).

Of the 5 welds of each adjustment that were carried out, 2 were intended for the tensile test with measurement of the percentage elongation, 2 were subjected to the bending test, and 1 was used for the metallographic test to carry out the count of the percentage of ferrite and austenite in the HAZ and in the ZF, as indicated in Figure 3c. The percentage of ferrite was determined using the color contrast method where a contrast is applied to the lightest phase, which is austenite, thus showing the percentage value of that phase in the image.

As the arc configuration depends on the peak base current values, the time of each, and the pulsation frequency, the adjustments proposed in Table 3 were determined through the following procedures:

- For analysis of the base and peak current values (Delta I):

The base and peak current values were relativized so that their arithmetic mean remained in all settings; however, the difference between them would be changed;

- For baseline and peak current time analysis (RC—Cyclic Ratio):

The time values were also relativized so that the value of this ratio is the percentage ratio of peak current time over base current time;

- For pulsation analysis (Frequency):

The value of the pulsation frequency in an exponential scale was changed to bring greater effectiveness in the analyses.

**Figure 4:**
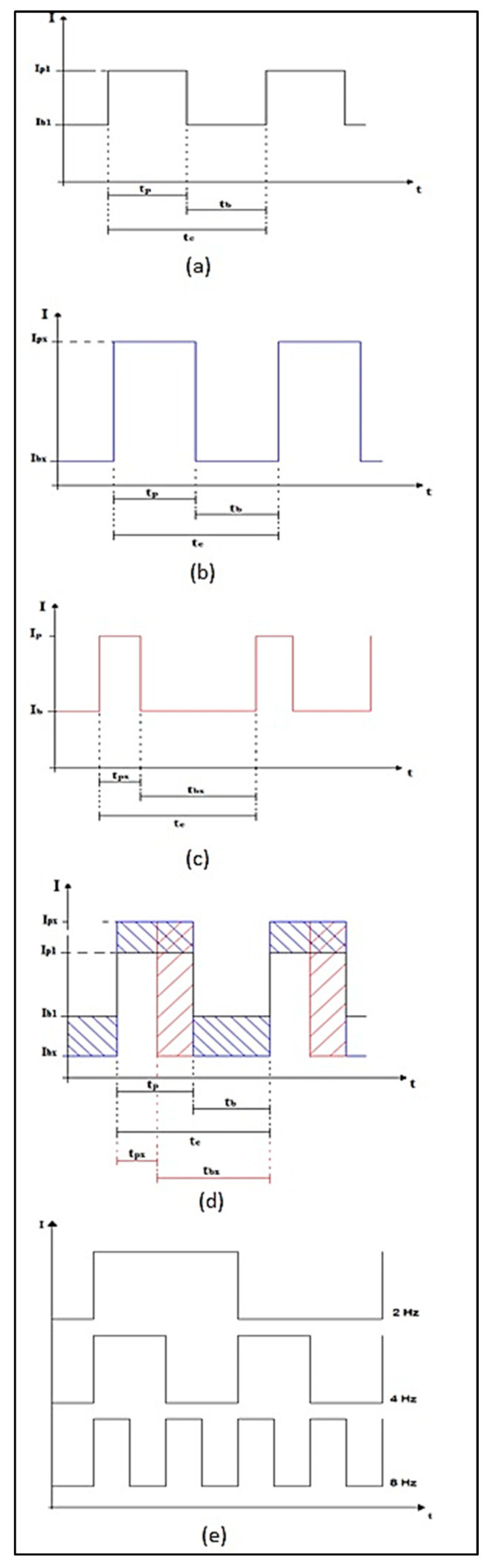
(**a**) Representation of the waveform of tuning number 17 (tuning with all parameters at their average values); (**b**) Representation of the maximum current variation during the experiments; (**c**) Representation of the maximum variation in the wave’s cyclical relation during the experiments; (**d**) Representation of waveform variation during experiments; (**e**) Representation of the pulse frequency variation.

Figure 4a illustrates the formatting of the pulsed arc while Figure 4b,c illustrates the maximum changes made and Figure 4d,e illustrates the formatting of these changes.

where:

Ip—Peak current or high current;
Ib—Base current or low current;
tc—Pulse time (period);
tp—Peak current time;
tb—Base current time.

## 3. Results and Discussion

The results are grouped and can be seen in Table 4.

**Figure 5:**
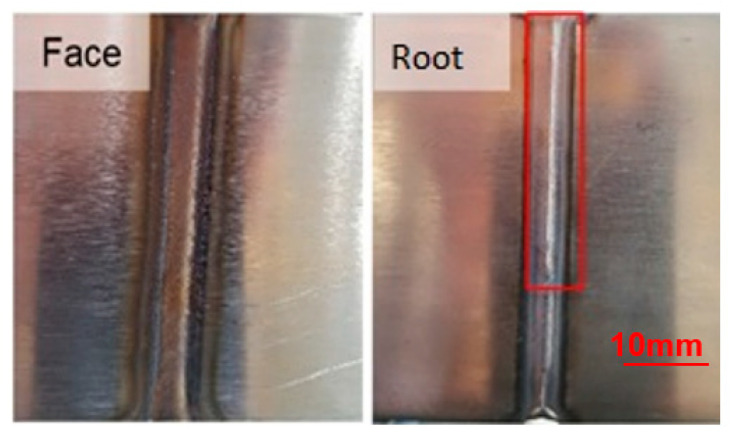
Weld without the appearance of fusion at the root (highlighted).

**Figure 6:**
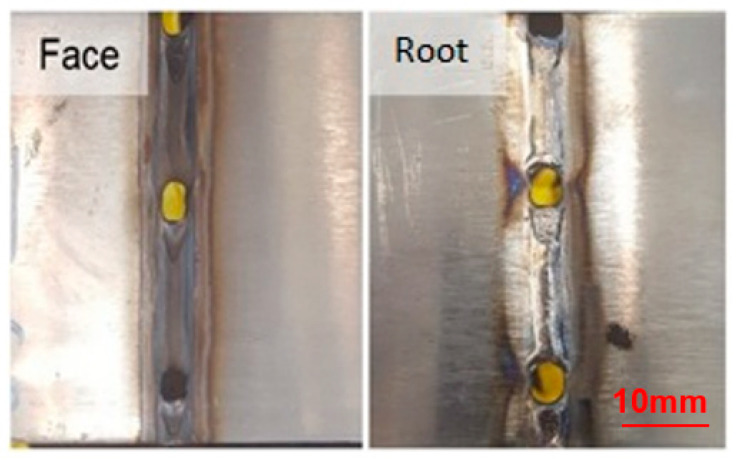
Weld with holes in the weld region.

**Figure 7:**
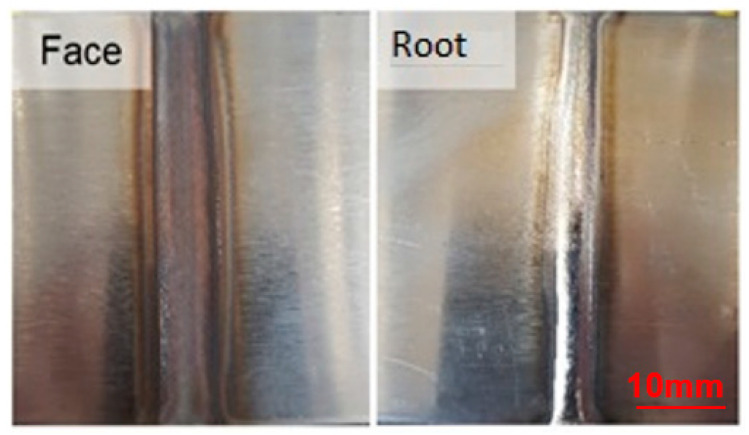
Weld with satisfactory visual appearance.

**Figure 8:**
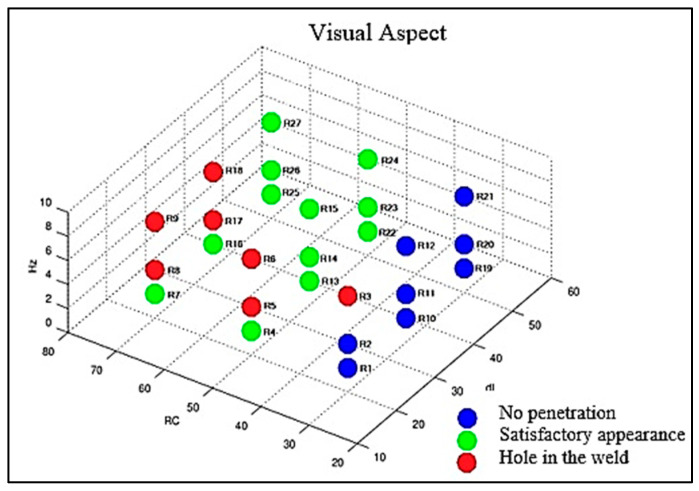
Compilation of visual appearance results.

The first criterion considered for the proposed analyses was the visual aspect, determined in Figure 5, Figure 6 and Figure 7, with its results compiled in the graph of Figure 8 so that one could optimize the evaluation process. The welds from the adjustments that did not reach a satisfactory visual appearance were discarded. To this end, the continuity of the weld bead was observed as well as the appearance of fusion at the root, which is the side of the tape opposite to exposure to the electric arc, indicating that the tape was welded in all its thickness. The welds from settings 1, 2, 10, 11, 12, 19, 20, and 21 did not have a root (Figure 5). The welds from settings 3, 5, 6, 8, 9, 17, and 18 showed holes in the weld path (Figure 6). The welds from the other adjustments presented a satisfactory visual appearance (Figure 7).

**Figure 9:**
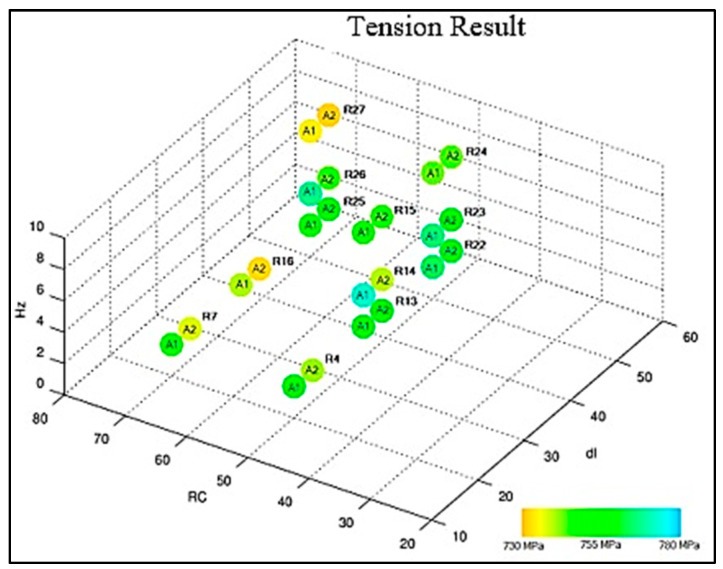
Compilation of tension results.

**Figure 10:**
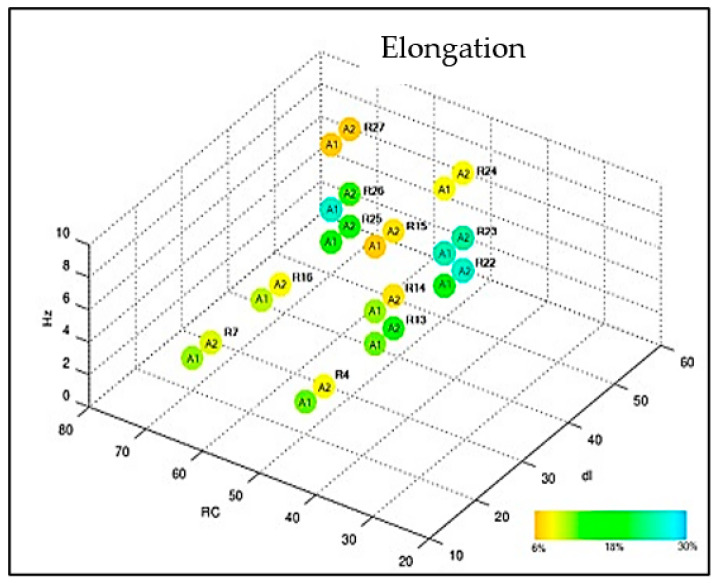
Compilation of elongation results.

Samples 1 and 2 of each adjustment were subjected to the tensile tests with measurement of percentage elongation. The weld performances were compared and presented in the graphs of Figure 9 and Figure 10. All samples subjected to the tensile test obtained satisfactory results when compared to the literature values (620 MPa). It is noted that the samples subjected to adjustment 23 obtained excellent tensile strength limit results: 768.35 MPa and 759.29 MPa associated with an elongation of 27% and 26%, respectively. The presented results agree with the findings of [14,25,26].

**Figure 11:**
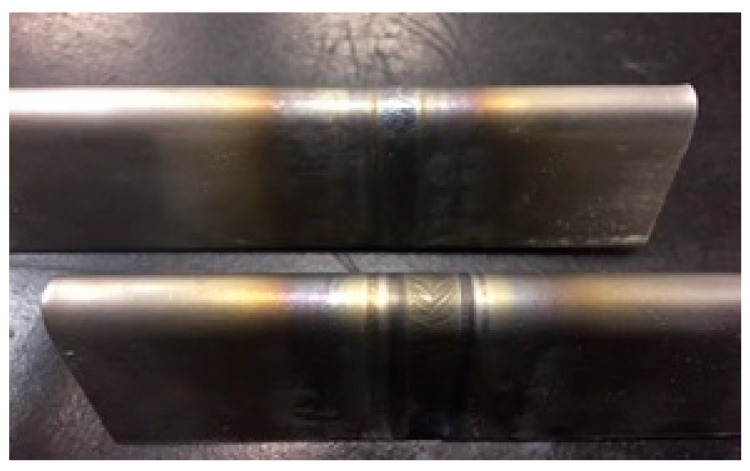
Bending view of the pulled face.

**Figure 12:**
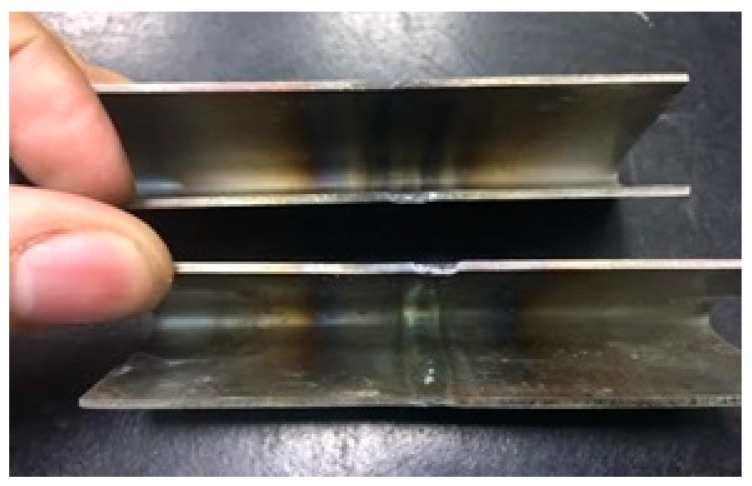
Bending view of the pulled root.

Samples 3 and 4 were subjected to bending tests according to the ASME IX standard [27]. This test plays a crucial role in evaluating and controlling the quality of materials as it allows evaluating ductility, that is, the material’s ability to plastically deform without fracturing. Furthermore, it allows checking the integrity of the weld, highlighting welding defects, such as cracks, inclusions, or porosities, which can compromise the strength and durability of the welded joints. With an additional connotation and eliminatory character, these tests results proved that all welds that performed with a satisfactory visual appearance were approved, as shown in Figure 11 and Figure 12.

**Figure 13:**
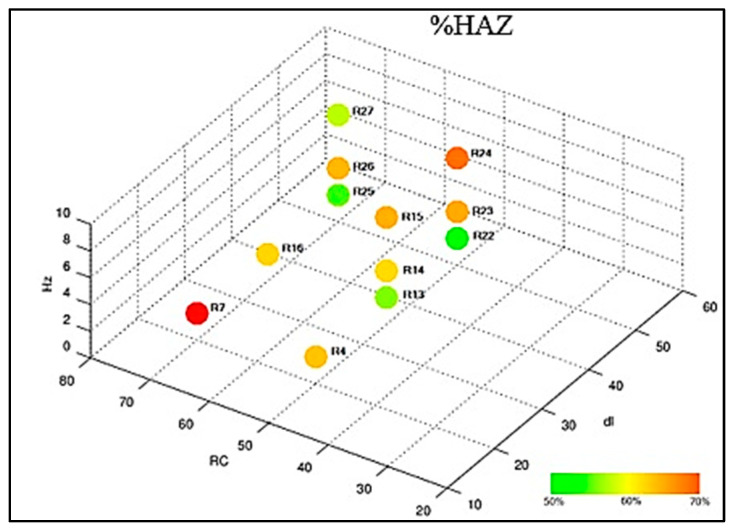
Compilation of ferrite count results in the HAZ.

**Figure 14:**
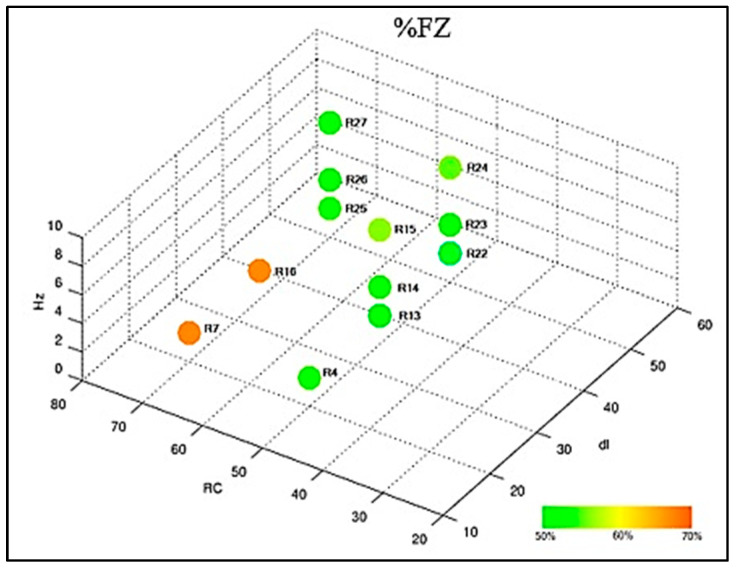
Compilation of ferrite count results at FZ.

Sample 5 was subjected to the metallographic test to count the percentage of ferrite in the HAZ and in the FZ. The performances of the settings are shown in the graph of Figure 13 and Figure 14. It was possible to observe that the region of the heat-affected zone (HAZ) has a percentage of ferrite higher than the fusion zone; this is due to the heating occurring from the ends to the center of the weld bead. The results corroborate with those of the authors’ [28,29,30].

In order to verify the influence of each variable, the analyses were performed in groups according to Table 5, where, among the three variables of interest, only one had its values changed. For example, for the study of frequency, nine groups were created with three adjustments each, where, in each group, the RC and Delta I values were maintained and the frequency values were changed [31].

### 3.1. Frequency Variation

It is possible to observe that with the increase in the pulse frequency, there is a random change in the tensile strength limit values and percentage elongation, an increase in the percentage values in the balance of ferrite and austenite, both in the HAZ and FZ. In addition, the appearance of the weld moves from an acceptable condition to a holed condition.

### 3.2. Variation in the Cyclic Ratio

It is possible to observe that with the increase in the cyclic ratio, there is a reduction in the tensile strength limit values and percentage elongation and an increase in the percentage values in the balance of ferrite and austenite, both in the HAZ and FZ. Moreover, the appearance of the weld moves from a no penetration condition to an acceptable condition.

### 3.3. Variation in Delta I

It is possible to observe that with the increase in the current delta, there is no significant alteration in the tensile strength limit values and percentage elongation; most of the time, these values alternate, making it impossible to determine a characteristic behavior. With regard to the balance, there is a reduction in the percentage values in the balance of ferrite and austenite, both in the HAZ and in the FZ. Furthermore, the appearance of the weld moves from a hole condition to an acceptable condition.

## 4. Conclusions

More important than getting an ideal adjustment for the pulsed arc setting in the TIG welding process is knowing how to achieve it. From the work conducted, it is possible to define which parameters will need to be adjusted in obtaining the desired results in a more agile and precise way, where the following can be concluded:

The changes with the cyclic relationship were more significant when considering the visual aspect of the weld.

The changes with the frequency were more significant when considering the mechanical property of percentage elongation.

The changes with the current Delta were more significant when considering the ferrite rate in the thermally affected zone.

For the property of limit voltage of tensile strength and ferrite rate in the molten zone region, no results were found that allow the linkage of these variations to the changes of each of the parameters.

## 5. Importance and Relevance

The present work brought results of paramount importance for the realization of welding on duplex stainless steel tape S31803. The knowledge of the variables associated with good welding quality provides greater robustness for the process, thus avoiding rework and financial losses during the production of the flexible duct.

## Figures and Tables

**Table 1:** Composition (% by weight) of the UNS S31803 duplex stainless steel strip in percentages [13].

Element	C	Mn	Si	Cr	Ni	P	S	Mo
%	0.03	2.0	1.0	21.0–23.0	4.5–6.5	0.03	0.02	2.5–3.5

**Table 2:** Mechanical properties of UNS S31803 duplex stainless steel [13].

UNS	σ_p0.2(MPa)_	σ_m(MPa)_	A%
S31803	450	620	25

**Table 3:** Adjustment proposals for the study itinerary.

Adjustment	Delta I (A)	CR (%)	Frequency (Hz)	Peak Current (A)	Base Current (A)	Torch Speed (mm/min)
1	20	30	2	180	36	250
2	20	30	4	180	36	250
3	20	30	8	180	36	250
4	20	50	2	180	36	250
5	20	50	4	180	36	250
6	20	50	8	180	36	250
7	20	70	2	180	36	250
8	20	70	4	180	36	250
9	20	70	8	180	36	250
10	35	30	2	160	56	250
11	35	30	4	160	56	250
12	35	30	8	160	56	250
13	35	50	2	160	56	250
14	35	50	4	160	56	250
15	35	50	8	160	56	250
16	35	70	2	160	56	250
17	35	70	4	160	56	250
18	35	70	8	160	56	250
19	50	30	2	144	72	250
20	50	30	4	144	72	250
21	50	30	8	144	72	250
22	50	50	2	144	72	250
23	50	50	4	144	72	250
24	50	50	8	144	72	250
25	50	70	2	144	72	250
26	50	70	4	144	72	250
27	50	70	8	144	72	250

**Table 4:** Results matrix.

Adjustment	Visual Aspect	S1		S2		S3	S4	S5	
		Tension (MPa)	Elong. (%)	Tension (MPa)	Elong. (%)	Bending		% Ferrite FZ	% Ferrite HAZ
1	Rootless	-	-	-	-	-	-	-	-
2	Rootless	-	-	-	-	-	-	-	-
3	Hole	-	-	-	-	-	-	-	-
4	Good	759.12	14	744.69	10	OK	OK	54.32	61.14
5	Hole	-	-	-	-	-	-	-	-
6	Hole	-	-	-	-	-	-	-	-
7	Good	753.15	13	740.52	11	OK	OK	63.14	67.04
8	Hole	-	-	-	-	-	-	-	-
9	Hole	-	-	-	-	-	-	-	-
10	Rootless	-	-	-	-	-	-	-	-
11	Rootless	-	-	-	-	-	-	-	-
12	Rootless	-	-	-	-	-	-	-	-
13	Good	758.84	14	758.41	16	OK	OK	52	56.46
14	Good	775.77	13	742.98	9	OK	OK	53.95	60.39
15	Good	752.41	8	750.30	9	OK	OK	56.54	61.83
16	Good	743.49	12	735.38	10	OK	OK	63.05	60.55
17	Hole	-	-	-	-	-	-	-	-
18	Hole	-	-	-	-	-	-	-	-
19	Rootless	-	-	-	-	-	-	-	-
20	Rootless	-	-	-	-	-	-	-	-
21	Rootless	-	-	-	-	-	-	-	-
22	Good	766.19	18	761.70	28	OK	OK	48.86	53.32
23	Good	768.35	27	759.29	26	OK	OK	53.51	62.02
24	Good	747.3	10	747.88	10	OK	OK	55.85	63.95
25	Good	753.08	16	756.77	16	OK	OK	54.42	55.60
26	Good	770.22	28	749.04	15	OK	OK	53.62	61.55
27	Good	737.29	8	734.57	8	OK	OK	53.64	57.60

**Table 5:** Definition of interest groups.

Frequency Study, Maintaining RC And Delta I									
Group	1	2	3	4	5	6	7	8	9
N° Regions	1,2 e 3	4, 5 e 6	7, 8 e 9	10, 11 e 12	13, 14 e 15	16, 17 e 18	19, 20 e 21	22, 23 e 24	25, 26 e 27
CR Study, Maintaining Frequency And Delta I									
Group	10	11	12	13	14	15	16	17	18
N° Regions	1, 4 e 7	2, 5 e 8	3, 6 e 9	10, 13 e 16	11, 14 e 17	12, 15 e 18	19, 22 e 25	20, 23 e 26	21, 24 e 27
Delta I Study, Maintaining RC And Frequency									
Group	19	20	2	22	23	24	25	26	27
N° Regions	1, 10 e 19	2, 11 e 20	3, 12 e 21	4, 13 e 22	5, 14 e 23	6, 15 e 24	7, 16 e 25	8, 17 e 26	9, 18 e 27

## Data Availability

Not applicable.
